# Association between Religious Beliefs and HPV Vaccination Attitudes among College Students

**DOI:** 10.3390/vaccines11101623

**Published:** 2023-10-21

**Authors:** Hannah Hittson, Leah McAleer, Lydia Saucedo, Lindsay Mahler, Gabriel Andino, Andie Zorba, Sarah Walden, Brett E. Pickett, Brian D. Poole, Erika L. Abel

**Affiliations:** 1 Honors Program, Honors College, Baylor University, Waco, TX 76798, USA; hannah.hittson@utsouthwestern.edu (H.H.); leah.mcaleer@utsouthwestern.edu (L.M.); lydia_saucedo1@baylor.edu (L.S.); lindsay_mahler1@baylor.edu (L.M.); gabriel.andino@utsouthwestern.edu (G.A.); andie_zorba1@baylor.edu (A.Z.); 2 Baylor Interdisciplinary Core, Honors College, Baylor University, Waco, TX 76798, USA; sarah_walden@baylor.edu; 3 Department of Microbiology and Molecular Biology, Brigham Young University, Provo, UT 84602, USA; brett_pickett@byu.edu (B.E.P.); brian_poole@byu.edu (B.D.P.)

**Keywords:** Human Papillomavirus, HPV vaccine, vaccine hesitancy, religious values, religious college students

## Abstract

Objective and Participants: The authors sought an updated examination of attitudes toward Human Papillomavirus (HPV) catch-up vaccination among college students at a private religious university. Methods: A total of 1557 college students completed a 62-question survey of religious and HPV vaccination attitudes during the fall of 2021. Students’ willingness to receive catch-up HPV vaccination and willingness to vaccinate a future child against HPV were recorded. Results: Of the 46.8% of students who reported being unvaccinated or unaware of vaccination status, ~26% reported being uninterested in receiving catch-up HPV vaccination; ~22% of all students surveyed reported being unwilling to vaccinate a future child against HPV. The strongest predictors of vaccine hesitancy included religious concerns about sexual abstinence and safety concerns. Conclusions: College health professionals can increase the rate of HPV vaccination among college students and subsequent future generations by addressing the safety and utility of the vaccine regardless of intentions for sexual abstinence prior to marriage. Additionally, rather than a uniform approach to all students who self-identify as Christian, an effort to identify and discuss the unique religiously influenced beliefs of individual students is recommended when discussing HPV vaccination.

## 1. Introduction

An estimated 43 million Americans are currently infected with Human Papillomavirus (HPV). With 13 million new cases expected annually, HPV is the most common sexually transmitted infection in the United States [1]. Between 50% to 80% of unvaccinated, sexually active adults are predicted to be infected with HPV at some point in their lives [2,3,4].

Among the 40 different HPV strains that are sexually transmitted, low-risk HPV types cause transient infections, such as genital skin warts. High-risk HPV infections can persist in lesions that may progress to cancers of the oral cavity, oropharynx, vulva, vagina, cervix, penis, or anus [5,6]. Oncogenic HPV strains 16 and 18 are responsible for ~90% of anal and cervical cancers, ~70% of vaginal and vulvar cancers, and ~60% of penile cancers [7]. HPV incidence is highest among teens and young adults [8]. Yet, studies suggest that fewer than 50% of college students are aware that HPV infection can progress to multiple types of cancer or that the HPV vaccine is effective in preventing these cancers [9,10,11].

Gardasil-9 is an FDA-approved vaccine that protects against 9 strains of HPV, including types 16 and 18. A complete HPV vaccination series prevents nearly 100% of HPV-associated cancers in males and females [12,13]. The Center for Disease Control’s Advisory Committee on Immunization Practices (ACIP) recommends initiating the vaccination series in children ages 9 to 12; a two-dose series is sufficient if initiated prior to age 15, while a three-dose series is recommended if initiated after age 15 [14].

Despite the clear benefit, HPV vaccination rates in the United States fall far below the Healthy People 2020 goal of 80%. As of 2019, only ~54% of adolescent girls and boys aged 13 to 17 were estimated to have completed the HPV vaccine series [15]. Similarly, only ~55% of college students report having received at least one dose of the vaccine [10,11,16,17,18]. Due to the documented deficiency in HPV vaccination coverage among college students (who are at the age of newly acquired independence regarding personal healthcare decisions and sexual activity), college healthcare providers and educators can play a significant role in cancer prevention through efforts to promote HPV vaccination.

Multiple reasons for parental non-adherence to the ACIP’s recommendation for HPV vaccination have been explored, including a lack of HPV and vaccine awareness, lack of provider recommendation, parental safety concerns, and concerns over perceived link to increased sexual activity [19,20,21,22,23]. Additionally, various studies have demonstrated a reduced uptake of the HPV vaccine in religious populations [24,25,26,27], and church affiliation and level of religious commitment have been associated with parental hesitancy to vaccinate children against HPV [27,28,29,30]. The idea that HPV vaccination promotes increased sexual activity, combined with a strong parental adherence to a faith tradition that encourages sexual abstinence prior to marriage, appears to contribute to negative parental attitude toward vaccination of their children.

While multiple studies have attempted to examine the reasons students arrive at college campuses unvaccinated, less attention has been given to robust examination of religious college student attitudes toward HPV “catch-up” vaccination (recommended for individuals who did not receive the first dose of the HPV vaccine prior to age 15, including college-aged students younger than 26). Even after the need for catch-up vaccination is noted, studies suggest that vaccine uptake among college students is minimal [10,31,32]. A study examining the 2010–2018 National Health Interview Survey found that only 4% of females and 3% of males initiated vaccination between the ages of 18 to 21 [33]. Further, in two studies of 209 and 256 female students offered free HPV vaccinations from university health clinics, only 28% and 5.5% completed the series [34,35].

A limited number of recent studies have suggested barriers to catch-up vaccination among college students, including fear of negative health consequences, perceived social influences, and fewer provider recommendations [10,31,32,36,37,38]. Literature further suggests a negative association between religiosity and HPV vaccine acceptance among college-aged women, emphasizing the importance of sexual purity in honor-endorsing women as a limiting factor in vaccine uptake [25,36]. Given the impact of social influencers (such as parents and close friends) on college students’ stances on vaccination and other healthcare decisions, we hypothesize that unvaccinated students raised in homes emphasizing specific religious values surrounding sexual behaviors are particularly resistant to catch-up HPV vaccination [18,39,40,41,42]. Therefore, a more refined understanding of the influence that religious beliefs have on a student’s willingness to receive a catch-up HPV vaccine or to vaccinate a future child against HPV is necessary and can be used to guide vaccine promotion efforts among religious college students. An updated view of vaccination attitudes is specifically desired given worldwide vaccination debates in the wake of COVID-19 vaccine mandates impacting churches and other organizations.

The goal of this study was to address these gaps in the published literature regarding religious college student attitudes toward the HPV vaccine with a focus on relationships between vaccination attitudes and specific religious beliefs and traditions. We aimed to characterize factors that influence willingness to receive catch-up HPV vaccination among students at a major Christian university. Then, based on the assumption that these college students are likely to become parents, healthcare providers, or community leaders, we further sought to characterize factors underlying resistance to vaccination of current or future children, specifically focusing on religiously influenced views regarding sexuality. Our results suggest that college health professionals and educators may play an influential role in promoting the HPV vaccine among college students by addressing common misconceptions about the vaccine, particularly among students struggling to reconcile religious values with perceived controversial healthcare decisions. The information provided by this study will aid healthcare professionals in discerning the best strategies for HPV vaccine promotion on college campuses, particularly on college campuses with a large proportion of religious students. However, a “one-size-fits-all-Christians” approach is not supported by our data; rather, health promotions should honor the variability in beliefs and attitudes among Christian students.

## 2. Materials and Methods

### 2.1. Survey Population and Recruitment

This cross-sectional study of vaccination attitudes was conducted from August to December 2021 among a convenience sample of college students at Baylor University, a private Baptist university in central Texas that enrolls a high proportion of religious students. A convenience sample was utilized due to logistical barriers to random sampling. The self-administered survey was approved by Baylor University’s Institutional Review Board and delivered using Qualtrics XM (Provo, UT, USA). Subject recruitment occurred through posted fliers and announcements by professors at Baylor University during the fall semester of 2021. To incentivize participation, professors offered extra credit in their courses for student participation. Faculty who advertised the survey were recruited through two methods: (1) contacting professors whom the undergraduate researchers knew and had a relationship with and (2) emailing professors of high undergraduate enrollment courses. Involved professors ranged in discipline from biology to business to philosophy. In most cases, the recruitment process occurred early in the semester, prior to when professors may have developed the trust relationship required to influence student responses on the survey regarding vaccination. Further, students were assured that the survey responses would be confidential to avoid response bias.

### 2.2. Survey Description

In addition to collecting demographic information, students were asked about their HPV vaccination status using the prompt “I have initiated or completed the HPV vaccine series.” The response options were “Yes, I have been vaccinated against HPV”, “No, I have not been vaccinated against HPV”, or “I do not know whether I have been vaccinated against HPV.” Students were also asked about openness to receiving the vaccine using the following question and response options: “The HPV vaccine is recommended for individuals under the age of 26. If you are unvaccinated (or potentially unvaccinated) and within this age range, would you agree to consider receiving the vaccine? You will not be contacted about receiving the vaccine if you answer yes” with response options: “Yes, I would consider receiving the HPV vaccine,” “I am not in this age range but I would consider getting the vaccine if I were,” “No, I would not consider receiving the HPV vaccine,” “I am not in this age range but would not consider the vaccine if I were,” or “I have already received the HPV vaccine.”

Respondents were also asked about personal religious beliefs and affiliations, opinions about vaccine safety, knowledge of HPV, HPV transmission, and the HPV vaccine. Where appropriate, responses were collected on a 5-point Likert scale. Individuals who did not progress through the entire survey, individuals under the age of 18, and individuals who were not undergraduate students at Baylor were removed from the data pool. Respondents were given the option to elect against answering questions due to the uncomfortable nature of the subject, and blank responses were replaced with the question’s average Likert response value during data analysis. Throughout the survey, various attention check questions (such as, *“This question wants to know if you agree that the moon is located in outer space. Please choose the answer that says, ‘neither agree nor disagree’”*) served to ensure attentive survey completion. Individuals who failed the attention checks were removed from the final survey population. The survey was open for four months, and in total, 1557 valid responses were collected from undergraduate students at Baylor.

### 2.3. Statistical Analysis

The primary outcome variables assessed were the students’ reported openness to vaccinate themselves (a measure of their attitude toward catch-up vaccination) and intent to vaccinate future children against HPV. Among unvaccinated students and students unaware of their vaccine status, attitudes toward catch-up vaccination were dichotomized into two responses: Catch-up Vaccine Accepting (including responses: *Yes, I would consider receiving the HPV vaccine* and *I am not in the recommended age range but I would consider getting the vaccine if I were)* and Catch-up Vaccine Resistant (including responses: *No, I would not consider receiving the HPV vaccine* and *I am not in the recommended age range but would not consider the vaccine if I were*). The intent to vaccinate future children against HPV was assessed using the following prompt: “*I am likely to vaccinate my children against HPV OR I have vaccinated my children against HPV*.” This outcome measure was divided into “Vaccine Accepting” (including students who answered *Strongly Agree* or *Agree*) and “Vaccine Hesitant” (including students who answered *Strongly Disagree, Disagree,* or *Neither Agree nor Disagree*).

Random forest analysis was then used to identify survey questions that were most associated with responses to two target questions. This was performed using version 4.7–1.1 of the random forest library within the R statistical software (R Core Team, version 4.2.2, R Foundation for Statistical Computing, Vienna, Austria, 2022; https://www.R-project.org/, accessed on 13 March 2023). Specifically, a table containing the responses to all questions across all participants was used as input to the random forest analysis. The Likert scale response data for the target question was then transformed into one of two categories based on whether the response was positive or negative. The data used to train the machine learning model consisted of a randomly selected 70% of participants, while responses from the remaining 30% of participants were then used to test the model. The number of trees generated in the random forest for the target question was 10,000, while the number of variables randomly sampled as candidates at each split was ~8, equal to the square root of the number of columns present in the input table. The mean decrease in Gini index values, which represents entropy/impurity of a feature/question, with larger values representing more features/questions that were most useful in classifying the data, were calculated for each feature/question in the input table. The output from this analysis consisted of a table with the quantitative data for each question on a separate row. All responses were 93.97% accurate at predicting responses to Q42 with an area under the receiver–operator characteristic curve of 97.18%.

Basic statistical analyses were additionally performed using JMP, Version 16 (JMP, Version 16, SAS Institute Inc., Cary, NC, 1989–2022). To follow up on responses that were identified as reliable predictors of vaccination hesitancy in the random forest analyses, student responses to individual questions were compared using Pearson’s Chi-squared test, where *p* < 0.05 was considered statistically significant. The Chi-squared test was employed to examine bivariate associations between HPV vaccination status or willingness to vaccinate future children against HPV and study variables including religious affiliation, safety concerns related to HPV, and general childhood vaccine safety.

## 3. Results

### 3.1. Descriptive Analysis of Study Population

The study sample consisted of 1557 responses from Baylor University undergraduate students, including 497 males and 1052 females aged 18–30 (Mean (M) age = 19.2; SD = 1.3; Table 1). The majority of respondents were white (60.9%) and self-identified as Christian (79.6%). Of the Christian students, 25.5% identified as Baptist and 3% as Evangelical Protestant, while 21.2% identified as Catholic and 14.4% as Mainline Protestant. An additional 32.8% identified as non-denominational Christians, and 3.2% identified as members of other Christian denominations. Moreover, 66.3% of religious students surveyed reported participating in religious activities at least 2 or more times per month (Table 1). The majority (73.2%) of students who took the survey reported a STEM (Science, Technology, Engineering, or Math, including pre-health studies) major as their course of study at Baylor; in addition, 67.2% of respondents reported a family household income level greater than 100,000 USD (Table 1).

### 3.2. Analysis of Indicators of HPV Catch-Up Vaccination Attitudes

Of the respondents, 53.2% reported having initiated or completed the HPV vaccine series, 21.1% reported not being vaccinated against HPV, and 25.7% were not aware of their vaccination status (Table 1). Our first goal was to characterize predictors of the total study population’s attitude toward catch-up HPV vaccination. A random forest machine learning model was employed to identify which survey questions best predicted stance toward the concept of student self-vaccination against HPV. Responses to the question: “The HPV vaccine is recommended for individuals under the age of 26. If you are unvaccinated (or potentially unvaccinated) and within this age range, would you agree to consider receiving the vaccine?” were examined. Those considered to have a negative stance toward catch-up vaccination were students who chose one of the following answers: (1) No, I would not consider receiving the HPV vaccine, or (2) I am not in this age range but would not consider the vaccine if I were. All other responses were considered indicative of a positive attitude toward vaccination. (Because students who reported prior HPV vaccination may have chosen it for themselves, these groupings were considered the least biased.) The results of the random forest analysis are summarized in (Table 2). Demonstrating the strength of modeling, the strongest predictors of openness to consideration of HPV catch-up vaccination were responses to questions asking the student’s stance toward vaccinating their potential future children against HPV and a question about the student’s own HPV vaccination history.

Strikingly, strong predictors of openness toward HPV catch-up vaccination were the responses to questions regarding the acceptability of sexual activity prior to marriage and the student’s adherence to religious teachings regarding sexual activity prior to marriage. Similarly, responses to questions regarding the utility of HPV vaccination for sexually abstinent individuals were predictive of stance toward catch-up vaccination, as were responses to questions about the safety of the HPV vaccine. These findings suggest that HPV catch-up vaccination outreach efforts on religious campuses will require efforts to explain the benefit and safety of HPV vaccination even if sexual abstinence is intended.

For catch-up intervention purposes, we considered the target audience for vaccination intervention strategies to be those students who did not report previous vaccination against HPV. This target population included students who were knowingly unvaccinated or unaware of their vaccination status. Of the students who did not report previous HPV vaccination, only 73.7% were open to considering HPV vaccination; the remaining 26.3% of the students were resistant to the idea of catch-up vaccination (Table 3). To determine whether predictors of stance toward catch-up vaccination detected in the random forest analyses persisted in the intervention target group, a series of Chi-squared analyses were conducted. We explored the directionality of the association between responses to multiple survey questions in the identified categories and the students’ stances toward catch-up vaccination against HPV, again using responses to the question: “The HPV vaccine is recommended for individuals under the age of 26. If you are unvaccinated (or potentially unvaccinated) and within this age range, would you agree to consider receiving the vaccine?”

Religious affiliation and religiously related viewpoints were significantly associated with unwillingness to explore HPV catch-up vaccination (Table 3). Although the population of unvaccinated students who were agnostic, atheist, or affiliated with other world religions was small, Christian students were significantly less likely to consider catch-up vaccination in comparison to non-Christian students (70.5% Christian students vs 87.1% non-Christian were open to considering HPV vaccination). Of note, the proportion of students who were hesitant toward HPV catch-up vaccination varied according to Christian tradition, ranging from 50% among mainline Protestant students to 38% among Baptist students to 18.8% among Catholic students, suggesting that Christian students are not uniform in their stance toward HPV vaccination. In general, more Christian students are open to catch-up vaccination than opposed; therefore, college health practitioners need not assume that a Christian patient will not be open to a recommendation for vaccination. To further explore why 30% of the population appears to be hesitant to consider HPV catch-up vaccination, we examined religious or religiously influenced beliefs more carefully.

In part, resistance to catch-up vaccination among religious students may be linked to the perception that the vaccine contains ingredients that conflict with religious beliefs. Among religious students with this concern, 65.1% were unwilling to consider receiving the vaccine. For a greater number of students, religiously related views regarding premarital sex appear to influence the choice to explore HPV catch-up vaccination. Those students who viewed premarital sex as unacceptable were significantly less open to considering catch-up vaccination in comparison to those who do not hold the same belief. Among religious students who expressed adherence to their religion’s teachings concerning sexual behavior, 34.6% reported hesitancy toward catch-up vaccination. Further, students who agreed or strongly agreed with the statement “People with diseases caused by HPV are responsible for their own suffering, because the virus is only transmitted through promiscuous sexual practices” were significantly less likely to be open to catch-up vaccination.

Data analysis further revealed associations between catch-up vaccination hesitancy and misconceptions about the utility of the HPV vaccine for those who abstain from sexual activity prior to marriage. Among the target population for HPV catch-up vaccination, 62.0% of students who agreed with the statement “If an individual abstains from sexual activity before marriage, they do not need to receive the HPV vaccine” were also unwilling to consider receiving the vaccine; by comparison, only 18.0% of students who do not hold this view were vaccination hesitant. Very similar results were obtained in response to the prompt “The HPV vaccine is not necessary if an individual has only one sexual partner in their lifetime.” These viewpoints held by Christian students who were resistant to catch-up vaccination suggest that they do not view themselves as currently at risk of HPV infection nor do they conceive of possible future exposures to HPV. The belief that those who do contract HPV are responsible for their own suffering suggests a reluctance to prevent the consequences of what they may consider sexual sin. Together, these responses illuminate the notion that hesitancy toward catch-up HPV vaccination may be rooted in naivete about the utility of the HPV vaccine despite sexual abstinence before marriage.

A lack of awareness about modes of HPV transmission was also noted among the study population, which may contribute to a false sense of safety. Out of all students surveyed, 96.1% of students correctly identified vaginal–penile sex as a mode of HPV transmission, but only 83.0% of students correctly identified oral sex (vaginal or penile) as a way to contract HPV (Table 4). Additionally, only 53.6% of students were aware that HPV can be passed between two individuals, even when a condom or dental dam is used. These data indicate that while most students were aware of HPV transmission through sexual intercourse, fewer knew that HPV could be transmitted through non-penetrative sexual behaviors or when sexual intercourse is protected. This has important implications for the advocacy of the HPV vaccine among religious college students who may embrace different definitions of sexual abstinence.

In addition to concerns regarding the utility of the vaccine among students who do not intend to have multiple sexual partners prior to marriage or more than one sexual partner for life, apprehensions about vaccine safety were also significantly associated with resistance toward the HPV vaccine (Table 2 and Table 3). A significantly large proportion of unvaccinated students who expressed belief the that the HPV vaccine is new and cannot be trusted or that the HPV vaccine has serious side effects reported hesitancy toward catch-up vaccination. Likewise, concerns that the vaccine contains dangerous toxins or a concern that vaccines have serious side effects were also noted. These findings suggest that reasons for HPV catch-up vaccination hesitancy are multifaceted, not solely rooted in religious beliefs about sexual purity, but also arising from concerns about the vaccine’s safety and efficacy as well as a lack of awareness regarding HPV viral transmission.

### 3.3. Analysis of Indicators of Hesitancy toward Vaccinating Future Children against HPV

Because parental or guardian choice determines whether children receive the HPV vaccine in the optimum window (ages 11–15), college health educators and professors possess a unique opportunity to influence future generations’ HPV vaccination rates nationwide by advocating for the HPV vaccine among current generations of college students. Design of outreach opportunities require an updated examination of attitudes toward the vaccination of children against HPV in a post-COVID society. For this reason, we also examined the attitudes of all students in the survey, regardless of their own HPV vaccination status, toward vaccination of current or future children. Interestingly, while 96.8% of students surveyed predicted vaccinating their current or future children with basic recommended childhood vaccines, only 77.7% predicted vaccinating children with the HPV vaccine, suggesting that a unique hesitancy toward the HPV vaccine exists.

**Figure 1:**
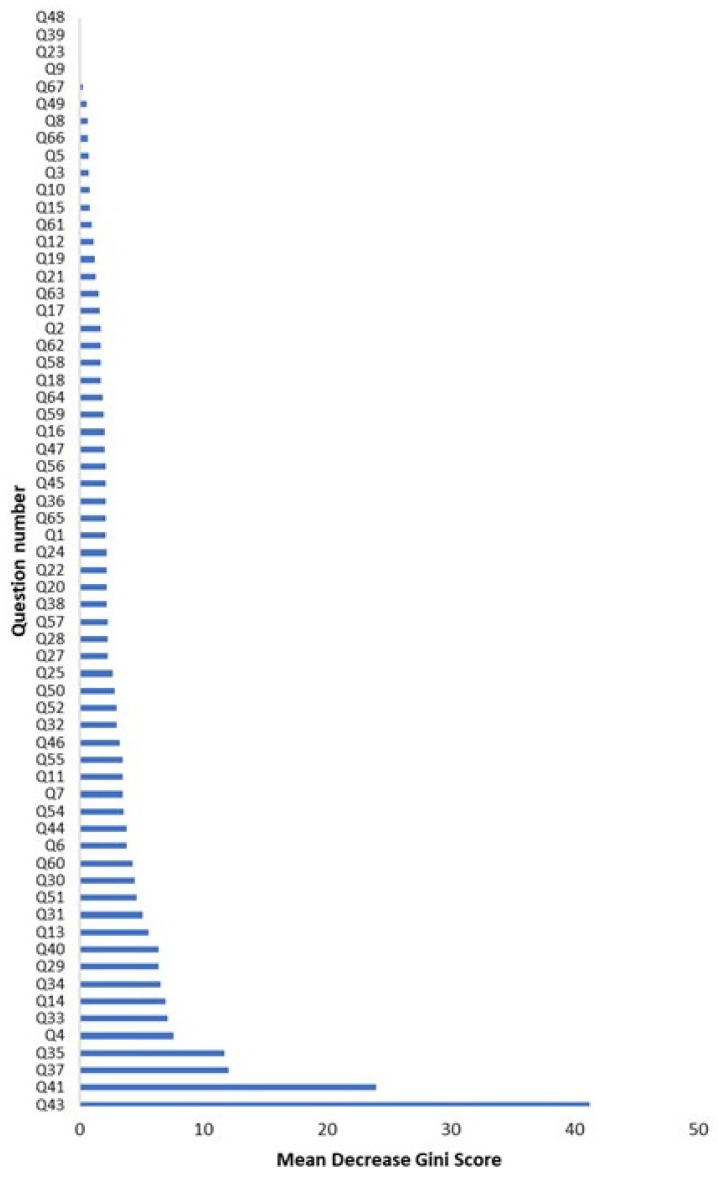
Results of random forest analysis. Random forest analysis was performed on the survey results, using the question “I am likely to vaccinate my children against HPV OR I have vaccinated my children against HPV” as the outcome. Higher mean decrease in the Gini Score shows as stronger predictors of the answer to this survey item. Questions 43 is the positive control. Question 41 (24, I have already initiated the HPV vaccine series) had the strongest predictive value, followed by 37 (12, If an individual abstains from sexual activity before marriage, they do not need to receive the HPV vaccine) and 35 (11.7, The HPV vaccine is not necessary if an individual has only had one sexual partner in life). All of the responses were 93.97% accurate at predicting responses to Q42 with an area under the receiver–operator characteristic curve of 97.18%.

To better characterize the basis for hesitancy toward vaccinating current or future children against HPV, a random forest machine learning model was used to identify which survey questions best predicted student response to the survey question “I am likely to vaccinate my children against HPV OR I have vaccinated my children against HPV.” The results of this random forest analysis are summarized in Figure 1. Underscoring the reliability of the model, a strong, positive association was found between openness toward a catch-up vaccination or previous vaccination against HPV and an intent to vaccinate future children. Of students who knew that they had previously received HPV vaccination, 96.3% also indicated an intent to vaccinate future children against HPV. In addition, of the students who reported being interested in a catch-up HPV vaccine, 74.9% also indicated an intent to vaccinate a future child against HPV.

Our modeling revealed multifactorial reasoning for resistance against vaccination of children against HPV. Similar to catch-up vaccination attitudes, predictive questions involving religious values and concerns regarding sexual behaviors were a striking theme. Other, somewhat less predictive questions addressed generalized vaccine efficacy and safety beliefs, safety and efficacy concerns specifically regarding the HPV vaccine, and other various social factors. Bivariate analyses were used to explore the directionality of the association between responses to questions in the identified categories and the students’ stances toward vaccinating current or future children against HPV.

### 3.4. Bivariate Analysis of Religious Participation and Stance toward Vaccinating Future Children against HPV

Because the random forest model indicated concerns regarding sexual purity prior to marriage as a strong predictor of negative HPV vaccine attitudes, the following sections pay particular attention to the religious beliefs of this student population. The associations between religious devotion and student attitudes toward vaccinating future children against HPV are shown in Table 5. As was true for catch-up vaccination attitudes, a greater proportion of Christian students disclosed hesitancy toward vaccinating a future child against HPV than non-Christian students (including those of other world religions, atheists, and agnostics. It should be noted that this was a diverse and not a particularly well-represented group within our study population, with only 316 non-Christian participants in total).

We noted that Christians are not uniform in their stance toward vaccination of children against HPV; the proportion of hesitant students of different denominations ranged from 16.4% to 34.2% (Table 5). Christian students who reported higher church attendance (attending eight or more religious activities per month) were more likely to express negative HPV vaccine attitudes compared to students who reported less frequent attendance at religious activities (Table 5). Over 95% of religious students indicated that their religion had teachings on sexual behaviors, and those who strictly adhered to their religion’s teachings were less likely to be accepting of the HPV vaccine for children. Further, 45.1% of religious students somewhat or strongly agreed that premarital sex is unacceptable, and 13.6% believe that people with diseases caused by HPV are responsible for their own suffering. As was noted with our analysis of attitudes toward catch-up vaccination, students with these beliefs were less likely to be open to vaccination of children against HPV.

### 3.5. Beliefs Concerning Sexual Activity and Administration of the HPV Vaccine to a Future Child

The nature of HPV as a sexually transmitted infection (STI) is regarded as a barrier to advocating for the HPV vaccine among religious populations. As found in the exploration of catch-up vaccination attitudes, religious beliefs concerning sexual behavior were predictive of negative HPV vaccine attitudes; however, specific misconceptions about the administration of the vaccine or a failure to acknowledge possible future HPV exposure scenarios are important avenues for exploration to guide future intervention campaigns for both catch-up vaccination of young adults and future vaccination of adolescents.

As found for catch-up vaccination, the results of random forest analysis indicated that responses to statements regarding sexual monogamy, such as “If an individual abstains from sexual activity prior to marriage, they do not need to receive the HPV vaccine” and “The HPV vaccine is not necessary if an individual only has one sexual partner in life” were strongly associated with HPV vaccination attitude (Table 6). Bivariate analysis confirmed this association (Table 7). Roughly 60% of all students who hold these beliefs expressed hesitancy to vaccinate children against HPV in comparison to only 17% of students who do not agree with these statements.

In contrast to deliberating the choice to self-vaccinate against HPV, prior studies have suggested that parental resistance to vaccinating their children is partly rooted in a misconception that vaccination against HPV contributes to earlier onset of sexual activity among adolescents. Random forest and bivariate analyses indicated that this concern was at play in our population as well. Students who agreed with statements such as “Vaccinating children against HPV will make them more likely to have premarital sex” or “The possibility of contracting HPV helps prevent premarital sex” reported a lower intent to vaccinate future children (Table 6). Strikingly, only 35.8% of students who agreed with the statement “Vaccinating 11–15-year-old children against HPV is unnecessary because children are not likely to be sexually active” were open to vaccinating children against HPV (in comparison to 81.7% of students who did not agree with the statement) suggesting a lack of awareness of the lifetime benefit of vaccinating adolescents before commencement of sexual activity.

### 3.6. Analysis of Other Concerns and Stance toward Vaccinating Future Children against HPV

In addition to concerns about the necessity of the HPV vaccine for sexually abstinent individuals, the random forest model further identified concerns over vaccine safety and efficacy to be predictive of HPV vaccination hesitancy with regard to vaccinating future children. The association between concerns about the safety and efficacy of the vaccine and students’ attitudes toward HPV vaccination for future children is shown in Table 8. Students who do not trust that vaccines are effective at preventing disease, free of toxins, more helpful than harmful, or adequately safety tested are less likely to express intent to vaccinate future children against HPV. In analyses of both catch-up vaccination attitudes and attitudes toward vaccinating future children, fear that the HPV vaccine is “relatively new and not highly tested, therefore it cannot be trusted” was predictive of hesitancy. Additional social factors were identified as being predictive for HPV vaccination of a future child, as shown in Table 8. STEM education (including pre-healthcare education) was only mildly associated with positive vaccination attitude, although the 20.9% of STEM students who expressed HPV vaccination hesitancy could be seen as a cause for concern. Finally, students do appear to respond to social cues when forming vaccination attitudes. Students who perceive that receiving the HPV vaccine would affect how they are viewed in their social circles held a dimmer view of vaccinating future children against HPV. Students who agreed with the statement “The HPV vaccine is not required for enrollment in all public middle schools, so it must not be beneficial or needed” were less likely to hold a positive view of their future intent to vaccinate children against HPV.

## 4. Discussion

Interventions to promote HPV awareness and HPV vaccination among college students can address two purposes: the short-term objective of encouraging catch-up vaccination and the long-term goal of shaping future parents’ stances regarding the vaccination of children against HPV. To guide vaccination campaigns on college campuses, particularly religious campuses where HPV vaccination hesitancy may be heightened, a clear and current view of student perceptions regarding the vaccine is required. In addition to providing a more refined view of Christian college students’ attitudes toward HPV vaccination, this study captures the attitudes of students whose education was dramatically impacted by the effects of the COVID-19 pandemic and COVID-19 vaccination policies. Our findings suggest that in addition to wariness surrounding general vaccine safety, many students possess misconceptions about the HPV vaccine that are related to beliefs about sexual behavior promoted in many Christian households. College health providers and educators are uniquely poised to address these concerns as well as any additional safety and efficacy misconceptions that might have been reinforced by COVID-19 vaccine debates. Importantly, however, practitioners will not be able to apply a uniform approach to all Christian students, nor can efforts be focused on a singular Christian tradition or denomination. Christian students of the same denomination vary in their stance toward the vaccine. Rather, the educator could explore specific beliefs about sexual activity and HPV transmission.

Approximately 50% of students surveyed in this study reported having initiated or completed the HPV vaccine series. Approximately one-quarter of the population (25.7%) was not aware of their HPV vaccination status, indicating that young adults have difficulty remembering their vaccination experience, due to its initiation in childhood and adolescence. This lack of knowledge about vaccination status presents a barrier for college healthcare providers, as a student may be unmotivated to pursue the HPV vaccine series if they do not know whether they have previously initiated or completed the series. Therefore, efforts by college healthcare providers to empower young adult students to explore their vaccination history may be particularly fruitful in promoting catch-up vaccinations, increasing the likelihood that an unvaccinated student might seek more information about the HPV vaccine and potentially initiate or re-initiate the vaccine series.

Of the 46.8% of students who either reported being unvaccinated or were unaware of their vaccination status, approximately 26.3% were not open to considering catch-up vaccination (Table 3). Consistent with past studies, we found that religious affiliation and commitment are associated with lower vaccine acceptance in catch-up vaccination and the intent to vaccinate a future child [25,27,28,30,36]. Results from both random forest and bivariate analyses suggest that the intent to receive catch-up HPV vaccination is lowest among students who share concerns about the necessity of the vaccine for sexually abstinent individuals.

In particular, students who hold the belief that the HPV vaccine is not necessary if an individual does have sex until marriage are less likely to indicate a willingness to initiate the HPV catch-up vaccine series. Similarly, embracing religious attitudes involving sexual purity is a strong negative predictor of the intent to vaccinate a future child against HPV. Therefore, while educators or healthcare providers for young adults may be mindful and attentive to the concerns of Christian students when promoting the HPV vaccine, they may be able to more quickly uncover sources of resistance to the HPV vaccine if a frank discussion of sexual behavior can be achieved. We assume that the resistance to HPV vaccination (both catch-up vaccination and vaccination of children) is caused, in part, by the belief that sexual contact will be avoided by Christian adolescents due to commitment to religiously motivated abstinence. This assumption is supported by the difference in attitude toward the HPV vaccine versus other childhood vaccinations. Only ~77% of the surveyed population predicted vaccinating with the HPV vaccine while over 96% of students predicted vaccinating future children with the other childhood vaccines.

For these reasons, efforts to explain the utility of the HPV vaccine may be more successful if focused on the importance of the HPV vaccine in protecting against multiple types of cancer or unexpected life circumstances, rather than asking a student to part with their devotion to their religious identity. Further, a conversation discussing the modes of transmission of HPV might be required or helpful. Religious adolescents may believe that they preserve “technical virginity” by only having oral sex instead of penile–vaginal sex. Because Christian adolescents are often taught to preserve their virginity until marriage, some believe that they can still participate in oral sex, without committing sexual immorality according to the Bible, and thus remain a “technical virgin.” Among our study population, 17.0% of students reported not knowing that HPV could be spread through oral sex (Table 4). Therefore, providing information about the various modes of transmission of HPV and the correlation between oral transmission of HPV and head and neck cancer may be particularly useful in advocating for the vaccine. Future studies will include assessing the impact of HPV transmission education (including consideration of unexpected life events) on hesitancy to vaccinate, which would also help establish a causative relationship between a lack of knowledge regarding HPV transmission and negative vaccination attitudes.

Together, these results suggest that a two-pronged approach to intervention among college students may be effective: (1) discussions about vaccine safety versus HPV-associated cancer risks and (2) carefully encouraging students to consider protecting themselves against unfortunate future events outside their control, such as infidelity or undetected prior HPV infection of their future spouse, could be beneficial in advocating for the HPV vaccine. Interventions that occur now, as the student is still developing identity as a citizen, professional, and religious follower, have the potential to shape adult choices about vaccinating children later in life. Examples of interventions that employ these context-specific approaches include: (a) an on-campus vaccination campaign designed with and implemented by students who espouse religious views that are otherwise similar to the target population or (b) parent focus groups with religious leaders of individual worshipping communities to discuss HPV vaccination in a sensitive but tailored way.

Empowering college students to learn more about how vaccines work and their processes for safety approval by the Food and Drug Administration (FDA) may result in future parents, community leaders, and healthcare providers who are more confident in their own assessment of the utility of a vaccine, thereby increasing long-term HPV vaccination rates in the US. Interestingly, 73.2% of our study population reported a degree in the STEM field, including pre-healthcare professions (Table 1). Among these students, who have been expected to have robust scientific education about HPV and the benefits of HPV vaccination, 20.9% expressed hesitancy toward HPV vaccination for a future child (Table 8). Together, these results strongly suggest that calls for the inclusion of vaccine education (including a discussion of the reasons for variable efficacy of the flu and COVID-19 vaccines) and the role of the FDA in oversight of vaccine safety testing, approval, and long-term monitoring are warranted.

## 5. Conclusions

In conclusion, our findings suggest that emphasis on education about the utility of the HPV vaccine despite devotion to particular religious practices is necessary. Focusing on the necessity of the vaccine for life-long protection against HPV and HPV-related cancers will serve to not only promote catch-up vaccination rates among college-aged individuals but further aid in increasing the vaccination rates of future generations. Extensive efforts must be made by healthcare providers to disentangle religious and safety concerns from the efficacy of the HPV vaccine. In addition, the reevaluation of the undergraduate curriculum, particularly in the STEM departments, regarding vaccine safety may be particularly urgent in the wake of the COVID-19 pandemic and subsequent political debates. Finally, partnerships to address HPV and cervical cancer awareness with sensitivity to religious beliefs among specific worshipping populations or student groups may be an effective and tailored way to increase HPV vaccination rates.

### Limitations

While we found faith-based beliefs and safety concerns to be indicators of acceptance of the HPV vaccine in the study population, multiple other factors may influence a person’s decision to vaccinate a child against HPV that were not examined. Other indicators not assessed in our survey might include concerns regarding personal autonomy, presence or absence of provider recommendation, extent of government mistrust, desire for “natural” living free of drugs or biologicals, and political affiliation. Further, students with the strongest level of mistrust of science or resistance to vaccination may have refused the survey; the views of these students are thus not reflected in our results. Additionally, our recruitment method (professors offering extra credit to students in their courses) may have introduced selection bias based on the professors’ motivations for participation or communicated opinions regarding vaccination. Finally, our findings are based on convenience sampling of one university in the geographical south and may not fully reflect the views of all religious college students in the US. For example, our student population includes low proportions of Muslim, Hindu, and Jewish students; it also included a very low number of students who associate with no religion. Therefore, reasons for vaccination resistance among atheist or agnostic students could not be determined.

As a cross-sectional study, this is unable to establish causation. However, understanding the correlates to HPV vaccine attitudes suggests areas where educational interventions may be effective. Further work is necessary to see if interventions in the identified correlations are effective at influencing vaccine attitudes.

## Figures and Tables

**Table 1:** Study population demographic characteristics.

Characteristic		
	n	%
Age (years)		
Mean Age + SD	19.2 + 1.3	-
Age Range	18–30	-
Gender		
Female	1052	67.7
Male	497	32.0
Preferred not to answer	6	0.4
Race/Ethnicity		
White	948	60.9
Asian	285	18.3
Hispanic/Latino	192	12.3
Black or African American	79	5.0
Other/Unknown	34	2.2
American Indian or Alaskan Native	8	0.5
Native Hawaiian or Pacific Islander	4	0.3
USA Region of Origin		
Southwest—AZ, NM, OK, TX	800	51.4
West—AK, CA, CO, HI, ID, MT, NV, OR, UT, WA, WY	291	18.7
Midwest—IA, IL, IN, KS, MI, MIN, MO, ND, NE, OH, SD, WI	158	10.2
Southeast—AL, AR, FL, GA, KY, LA, MS, NC, SC, TN, VA, WV	124	8.0
Northeast—CT, DC, DE, MA, MD, ME, NH, NJ, NY, PA, RI, VT	79	5.1
Other	105	6.7
Academic Area of Study		
Non-STEM	417	26.8
STEM	1140	73.2
Average Family Income		
<10,000 USD	31	2.0
10,000–39,999 USD	72	4.7
40,000–69,999 USD	171	11.1
70,000–99,999 USD	231	15.0
>100,000 USD	1035	67.2
Religion		
Christian	1236	79.6
Other World Religions	172	11.1
Atheist/Agnostic	144	9.3
Christian Denomination Affiliation		
Mainline Protestant	177	14.4
Evangelical Protestant	37	3.0
Baptist	314	25.5
Catholic	262	21.2
Non-denominational	404	32.8
Other	39	3.2
Relationship Status		
In a relationship with another individual	466	29.9
Married	10	0.6
Single	1081	69.4
HPV Vaccination Status		
Vaccinated	828	53.2
Not Vaccinated	328	21.1
Unknown	400	25.7
Church Attendance/Religious Activity Participation (times per month)		
0	348	24.8
<1	124	8.8
2–4	462	32.9
5–8	306	21.8
>8	163	11.6

**Table 2:** Random forest model of predictors of openness to catch-up vaccination of self against HPV. Survey prompts that were most predictive of vaccination attitude are listed according to category of concern.

Religion or religiously related values
I strictly adhere to my religion’s teachings concerning sexual behavior Premarital sex is unacceptable
Views regarding HPV administration and indications
If an individual abstains from sexual activity prior to marriage, they do not need to receive the HPV vaccine
The HPV vaccine is not necessary if an individual has only one sexual partner in life
Vaccinating 11–15-year-old children against HPV is unnecessary because children are not likely to be sexually active
Views regarding the efficacy or safety of the HPV vaccine
The HPV vaccine is not required for enrollment in all public middle schools, so it must not be beneficial or needed The HPV vaccine is relatively new and has not been highly tested; therefore, it cannot be trusted
Other social, ethical, or cultural factors
If getting the HPV vaccine would protect my significant other from HPV and HPV-related cancers, I would consider receiving the vaccine
Which category best describes your parents’ (or guardian’s) yearly household income before taxes I am likely to vaccinate my children against HPV I have initiated or completed the HPV vaccine series

**Table 3:** Catch-up vaccination attitudes among students who did not report prior vaccination against HPV: association with religious beliefs and vaccine safety concerns.

Characteristic	Catch-Up Vaccination Hesitant		Catch-Up Vaccination Accepting		Total N		*p* Value
	*n* = 189		*n* = 529		*n* = 718		
	*n*	%	*n*	%	*n*	%	X^2^
Students’ openness to considering catch-up vaccination	189	26.3	529	73.7	718	100	-
Which category best describes your parents’ (or guardians’) yearly household income before taxes?							
<100,000 USD	80	29.4	192	70.6	272	38.3	0.09
≥100,000 USD	104	23.7	335	76.3	439	61.7	
Religion							<0.0001
Christian	170	29.5	406	70.5	576	80.6	
Other World Religion, Agnostic/Atheist	18	13.0	121	87.1	112	19.4	
Christian Denomination Affiliation							<0.0001
Mainline Protestant	10	50.0	10	50.0	20	3.5	
Evangelical Protestant	15	21.7	54	78.0	69	12.0	
Baptist	62	38.3	100	61.7	162	28.1	
Catholic	21	18.8	91	81.2	112	19.4	
Non-denominational	56	29.2	136	70.8	192	33.3	
Other	6	28.6	15	71.4	21	3.7	
Among religious students: The HPV vaccine contains ingredients that conflict with my religious beliefs.							
Strongly/Somewhat Agree	43	65.1	23	34.9	66	10.1	<0.0001
Neither Agree nor Strongly/Somewhat Disagree	137	23.3	451	76.7	588	89.9	
Premarital sex is unacceptable.							
Strongly/Somewhat Agree	143	41.8	199	58.2	342	47.7	<0.0001
Neither Agree nor Strongly/Somewhat Disagree	46	12.3	329	87.7	375	52.3	
Among students whose religion has teachings regarding sexual behavior: I strictly adhere to my religion’s teachings concerning sexual behavior.							
Strongly/Somewhat Agree	160	34.6	303	65.4	463	74.4	<0.0001
Neither Agree nor Strongly/Somewhat Disagree	13	8.2	146	91.8	159	25.6	
People with diseases caused by HPV are responsible for their own suffering because the virus is only transmitted through promiscuous sexual practices.							
Strongly/Somewhat Agree	49	47.1	55	52.9	104	14.5	<0.0001
Neither Agree nor Strongly/Somewhat Disagree	140	22.8	473	77.2	613	85.5	
The HPV vaccine is not necessary if an individual has only one sexual partner in their lifetime.							
Strongly/Somewhat Agree	83	62.9	49	37.1	132	18.4	<0.0001
Neither Agree nor Strongly/Somewhat Disagree	106	18.1	479	81.9	585	81.6	
If an individual abstains from sexual activity before marriage, they do not need to receive the HPV vaccine.							
Strongly/Somewhat Agree	85	62.0	52	38.0	137	19.1	<0.0001
Neither Agree nor Strongly/Somewhat Disagree	104	18.0	475	82.0	579	80.9	
HPV vaccine has serious side effects.							
Strongly/Somewhat Agree	61	47.7	67	52.3	128	17.9	<0.0001
Neither Agree nor Strongly/Somewhat Disagree	127	21.6	462	78.4	589	82.2	
The HPV vaccine is relatively new and not highly tested, therefore it cannot be trusted.							
Strongly/Somewhat Agree	48	64.9	26	35.1	74	10.3	<0.0001
Neither Agree nor Strongly/Somewhat Disagree	140	21.8	503	78.2	643	89.7	
Vaccines often have serious side effects.							
Strongly/Somewhat Agree	70	37.2	118	62.8	188	26.2	<0.0001
Neither Agree nor Strongly/Somewhat Disagree	119	22.5	411	77.6	530	73.8	
Vaccines are effective at preventing disease.							
Strongly/Somewhat Agree	155	23.3	511	76.7	666	92.8	<0.0001
Neither Agree nor Strongly/Somewhat Disagree	34	64.4	18	34.6	52	7.2	
Vaccines contain dangerous toxins.							
Strongly/Somewhat Agree	43	55.8	34	44.2	77	10.7	<0.0001
Neither Agree nor Strongly/Somewhat Disagree	146	22.8	495	77.2	641	89.3	
Vaccines are more helpful than harmful.							
Strongly/Somewhat Agree	134	21.4	493	78.6	627	86.8	<0.0001
Neither Agree nor Strongly/Somewhat Disagree	59	62.1	36	37.9	95	13.2	
The HPV vaccine is not required for enrollment in all public middle schools, so it must not be beneficial or needed.	22	66.7	11	33.3	33	4.6	<0.0001
Strongly/Somewhat Agree							
Neither Agree nor Strongly/Somewhat Disagree	167	24.5	516	75.6	683	95.4	
If getting the HPV vaccine would protect my significant other from HPV and HPV-related cancers, I would consider receiving the vaccine.							
Strongly/Somewhat Agree	111	20.7	425	79.3	536	74.7	<0.0001
Neither Agree nor Strongly/Somewhat Disagree	78	42.9	104	57.1	182	25.4	

**Table 4:** Student awareness of methods of HPV transmission.

Method of Transmission	Indicated Awareness		No Indicated Awareness	
	*n* = 1559			
	*n*	%	*n*	%
Vaginal–penile sex	1498	96.1	61	3.9
Penile–anal sex	1357	87.0	202	13.0
Penile–oral sex	1295	83.1	264	16.9
Vaginal–oral sex	1294	83.0	265	17.0
Sex even when condoms and/or dental dams are used	836	53.6	723	46.4
Sharing of hairbrushes	92	5.9	1467	94.1

**Table 5:** Attitudes toward vaccinating children against HPV among religious students: association with religion and religious teachings.

Characteristic	Vaccination Hesitant		Vaccination Accepting		Total N		p Value
	*n* = 330		*n* = 1073		*n* = 1403		
	*n*	%	*n*	%	*n*	%	X^2^
Religious Preference							
Christian	305	24.8	927	75.2	1232	87.8	<0.0001
Other World Religions	25	14.7	146	85.3	171	12.1	
Christian Denomination Affiliation							
Mainline Protestant	29	16.4	148	83.6	176	14.4	<0.0001
Evangelical Protestant	12	32.4	25	67.6	37	3.0	
Baptist	101	32.3	212	67.7	313	25.5	
Catholic	50	19.2	211	80.8	261	21.2	
Non-denominational	100	24.8	303	75.2	403	32.8	
Other	13	34.2	25	65.8	38	3.1	
Church Attendance/Religious Activity Participation (times per month)							
0	55	15.8	293	84.2	348	24.8	<0.0001
<1	23	18.6	101	81.5	124	8.8	
2–4	95	20.6	367	79.4	462	32.9	
5–8	96	31.4	210	68.6	306	21.8	
>8	61	37.4	102	62.6	163	11.6	
The HPV vaccine contains ingredients that conflict with my religious beliefs.							
Strongly/Somewhat Agree	56	68.3	36	31.7	92	5.9	<0.0001
Strongly/Somewhat Disagree	270	20.6	1043	79.4	1313	94.1	
Premarital sex is unacceptable.							
Strongly/Somewhat Agree	220	34.9	410	65.1	630	45.1	<0.0001
Neither Agree nor Strongly/Somewhat Disagree	110	14.3	658	85.7	768	54.9	
People with diseases caused by HPV are responsible for their own suffering because the virus is only transmitted through promiscuous sexual practices.							
Strongly/Somewhat Agree	59	30.9	123	69.1	191	13.6	0.013
Neither Agree nor Strongly/Somewhat Disagree	271	22.4	938	77.6	1209	86.4	
Among students whose religion has teachings regarding sexual behavior: I strictly adhere to my religion’s teachings concerning sexual behavior.							
Strongly/Somewhat Agree	262	28.6	653	71.4	915	71.2	<0.0001
Neither Agree nor Strongly/Somewhat Disagree	48	12.9	323	87.1	371	28.9	

**Table 6:** Attitudes toward vaccinating children against HPV among all students surveyed: association with beliefs concerning utility of administration of the HPV vaccine and impacts on sexual activity.

Characteristic	Vaccination Hesitant		Vaccination Accepting		Total N		*p* Value
	*n* = 346		*n* = 1207		*n* = 1553		
	*n*	%	*n*	%	*n*	%	X^2^
Vaccinating children against HPV sends them mixed messages about the acceptability of sexual activity.							
Strongly/Somewhat Agree	111	60.3	73	39.4	184	11.9	<0.0001
Neither Agree nor Strongly/Somewhat Disagree	234	17.1	1132	82.9	1366	88.1	
Vaccinating children against HPV will make them more likely to have premarital sex.							
Strongly/Somewhat Agree	73	57	55	43	128	8.3	<0.0001
Neither Agree nor Strongly/Somewhat Disagree	273	19.2	1150	80.8	1423	91.8	
If an individual abstains from sexual activity prior to marriage, they do not need to receive the HPV vaccine.							
Strongly/Somewhat Agree	106	60.2	70	39.8	167	10.9	<0.0001
Neither Agree nor Strongly/Somewhat Disagree	239	17.4	1132	82.6	1371	89.1	
The HPV vaccine is not necessary if an individual has only one sexual partner in life.							
Strongly/Somewhat Agree	104	59.4	71	40.6	175	11.3	<0.0001
Neither Agree nor Strongly/Somewhat Disagree	242	17.6	1131	82.4	1373	88.7	
The possibility of contracting HPV helps prevent premarital sex.							
Strongly/Somewhat Agree	124	27	340	73	466	30.1	0.0034
Neither Agree nor Strongly/Somewhat Disagree	219	20.2	865	79.8	1084	69.9	
Among students whose religion has teachings on sexual behavior: Because HPV is sexually transmitted, my family’s values will protect my children from contracting HPV.							
Strongly/Somewhat Agree	201	26.1	568	73.8	769	60.6	0.0295
Neither Agree nor Strongly/Somewhat Disagree	106	20.8	403	79.1	509	39.4	
Vaccinating 11–15-year-old children against HPV is unnecessary because children are not likely to be sexually active.							
Strongly/Somewhat Agree	88	64.2	49	35.8	137	8.8	0.0014
Neither Agree nor Strongly/Somewhat Disagree	258	18.3	1155	81.7	1413	91.2	
Because only females are at risk of developing cancer following HPV infection, males do not need to consider being vaccinated against HPV.							
Strongly/Somewhat Agree	6	42.9	8	57.1	14	0.9	0.0861
Neither Agree nor Strongly/Somewhat Disagree	340	22.1	1196	77.9	1536	99.1	
Women, but not men, can experience health problems after HPV infection.							
Strongly/Somewhat Agree	88	22.3	307	77.7	395	25.5	0.9973
Neither Agree nor Strongly/Somewhat Disagree	257	22.3	897	77.7	1154	74.5	

**Table 7:** Attitudes toward vaccinating children against HPV among all students surveyed: association with vaccine safety and efficacy concerns.

Characteristic	Vaccination Hesitant		Vaccination Accepting		Total N		*p* Value
	*n* = 346		*n* = 1207		*n* = 1553		
	*n*	%	*n*	%	*n*	%	X^2^
Vaccines are effective at preventing disease.							
Strongly/Somewhat Agree	294	19.9	1186	80.1	1480	95.3	<0.0001
Neither Agree nor Strongly/Somewhat Disagree	52	71.2	21	28.8	73	4.7	
Vaccines are more helpful than harmful.							
Strongly/Somewhat Agree	259	18.5	1143	81.5	1402	90.3	<0.0001
Neither Agree nor Strongly/Somewhat Disagree	87	58	63	42	150	9.7	
The HPV vaccine is relatively new and has not been highly tested; therefore, it cannot be trusted.							
Strongly/Somewhat Agree	60	71.4	24	28.6	84	5.4	<0.0001
Neither Agree nor Strongly/Somewhat Disagree	285	19.5	1179	80.5	1464	94.6	
HPV vaccine has serious side effects.							
Strongly/Somewhat Agree	92	49.5	94	50.5	186	12.0	<0.0001
Neither Agree nor Strongly/Somewhat Disagree	253	18.5	1112	81.5	1365	88.0	
Vaccines often have serious side effects.							
Strongly/Somewhat Agree	115	42.4	156	57.6	271	17.5	<0.0001
Neither Agree nor Strongly/Somewhat Disagree	231	18.0	1050	82.0	1281	82.5	
Vaccines contain dangerous toxins.							
Strongly/Somewhat Agree	59	58.4	42	41.6	101	6.5	<0.0001
Neither Agree nor Strongly/Somewhat Disagree	287	19.8	1162	80.2	1449	93.5	
I am likely to fully vaccinate my children with the basic recommended childhood vaccines (do not consider HPV vaccine in your response).							
Strongly/Somewhat Agree	307	20.4	1296	79.6	1503	96.8	<0.0001
Neither Agree nor Strongly/Somewhat Disagree	39	79.6	10	20.4	49	3.2	

**Table 8:** Attitudes toward vaccinating children against HPV among all students surveyed: association with other social factors.

Characteristic	Vaccination Hesitant		Vaccination Accepting		Total N		*p* Value
	*n* = 346		*n* = 1207		*n* = 1553		
	*n*	%	*n*	%	*n*	%	X^2^
In what region of the United States did you spend most of your childhood?							
Southwest—AZ, NM, OK, TX	170	21.3	628	78.7	798	51.4	<0.0001
West—AK, CA, CO, HI, ID, MT, NV, OR, UT, WA, WY	67	23.1	223	76.9	290	18.7	
Midwest—IA, IL, IN, KS, MI, MIN, MO, ND, NE, OH, SD, WI	35	22.3	122	77.7	157	10.1	
Southeast—AL, AR, FL, GA, KY, LA, MS, NC, SC, TN, VA, WV	21	16.9	103	83.1	124	8.0	
Northeast—CT, DC, DE, MA, MD, ME, NH, NJ, NY, PA, RI, VT	22	27.9	57	72.2	79	5.1	
Other	31	29.5	74	70.5	105	6.8	
Academic Area of Study							
Non-STEM	82	28.2	209	71.8	291	18.7	0.0086
STEM	264	20.9	998	79.1	1262	81.3	
Which category best describes your parents’ (or guardians’) yearly household income before taxes?							
<100,000 USD	134	26.6	369	73.4	503	32.7	0.0037
≥100,000 USD	207	20.0	827	80.0	1034	67.3	
I have initiated or completed the HPV vaccine series.							
Yes, Previously Vaccinated	31	3.7	797	96.3	828	53.3	<0.0001
No, Not Vaccinated/Unaware of Vaccination Status	315	43.5	410	56.6	725	46.7	
Among students who are unvaccinated or unaware of their vaccination status: Would you agree to consider receiving the HPV vaccine?							
Strongly/Somewhat Agree	133	25.1	396	74.9	529	73.7	<0.0001
Neither Agree nor Strongly/Somewhat Disagree	180	96.2	9	4.8	189	26.3	
Receiving the HPV vaccine will affect how people in my social circle will view me.							
Strongly/Somewhat Agree	37	52.9	33	47.2	70	4.5	<0.0001
Neither Agree nor Strongly/Somewhat Disagree	309	20.9	1173	79.2	1482	95.5	
The HPV vaccine is not required for enrollment in all public middle schools, so it must not be beneficial or needed.							
Strongly/Somewhat Agree	26	54.2	22	45.8	48	3.1	<0.0001
Neither Agree nor Strongly/Somewhat Disagree	320	21.3	1180	78.7	1500	96.9	

## Data Availability

Raw data are available upon request from the corresponding author.
